# The mitochondrial genome of *Caenis* sp. (Ephemeroptera: Caenidae) and the phylogeny of Ephemeroptera in Pterygota

**DOI:** 10.1080/23802359.2018.1467239

**Published:** 2018-05-15

**Authors:** Yin-Yin Cai, Ya-Jie Gao, Le-Ping Zhang, Dan-Na Yu, Kenneth B. Storey, Jia-Yong Zhang

**Affiliations:** a College of Chemistry and Life Science, Zhejiang Normal University, Jinhua, Zhejiang Province, China;; b Key Lab of Wildlife Biotechnology, Conservation and Utilization of Zhejiang Province, Zhejiang Normal University, Jinhua, Zhejiang Province, China;; c Department of Biology, Carleton University, Ottawa, Canada

**Keywords:** Ephemeroptera, Palaeoptera, Metapterygota, mitochondrial genome, phylogeny

## Abstract

The phylogenetic relationship between Ephemeroptera (mayflies) and Odonata (dragonflies and damselflies) remains hotly debated in the insect evolution community. We sequenced the complete mitochondrial genome of *Caenis* sp. (Ephemeroptera: Caenidae) to discuss the phylogenetic relationship of Palaeoptera. The mitochondrial genome of *Caenis* sp. is a circular molecule of 15,254 bp in length containing 37 genes (13 protein-coding genes, 22 tRNAs, and 2 rRNAs), which showed the typical insect mitochondrial gene arrangement. In BI and ML phylogenetic trees using 71 species of 12 orders, our results support the Ephemeroptera as the basal group of winged insects.

The phylogenetic relationships among the Ephemeroptera (mayflies), Odonata (dragonflies and damselflies) and Neoptera has been hotly debated by researchers, often based on their use of different molecular methods (Zhang et al. 2008; Regier et al. 2010; Ishiwata et al. 2011; Thomas et al. 2013). These researchers have proposed three main hypotheses, the Palaeoptera hypothesis ((Ephemeroptera + Odonata) + Neoptera) (Blanke et al. 2012), the basal Ephemeroptera hypothesis (Ephemeroptera + (Odonata + Neoptera)) (Zhang et al. 2008), and the basal Odonata hypothesis (Odonata + (Ephemeroptera + Neoptera)) (Li et al. 2014). Different hypotheses, using different datasets, genes or phylogenetic approaches, have received distinct support (Mallatt and Giribet 2006; Meusemann et al. 2010; Song et al. 2016). Compared to 42 families in Ephemeroptera, there are only 19 complete or nearly complete mitochondrial genomes of Ephemeroptera belonging to 11 families on Genbank (Tang et al. 2014; Gao et al. 2018). In this study, we sequenced the complete mitochondrial genome of another ephemeropteran species, *Caenis* sp. (MG910499), to provide more molecular data that enables researchers to further discuss three main hypotheses of the origin of winged insects.

The sample of *Caenis* sp. was collected in Tianmu Mountain (30°21′35′′ N, 119°26′12′′ E), China, identified by Dr. JY Zhang (College of Life Sciences and Chemistry, Zhejiang Normal University). Total genomic DNA was extracted from individual tissues of the sample using Ezup Column Animal Genomic DNA Purification Kit (Sangon Biotech Company, Shanghai, China). All mayfly samples and DNA samples were stored in the lab of Dr. JY Zhang. Universal primers were used to amplify some partial fragments as described in Zhang et al. (2008).

The complete mitochondrial genome of *Caenis* sp. was a typical circular DNA molecule of 15,254 bp in length containing 37 genes (13 protein-coding genes [PCGs]), 22 tRNAs, and two rRNAs) and an A + T region. The AT content of the whole genome was 68.4% and the length of the control region was 527 bp with 71.6% AT content.

The phylogenetic relationship was constructed from the 13 PCGs by following the Bayesian Inference (BI) and Phylip Maximum Likelihood (PhyML) methods and using MrBayes version 3.2 (Ronquist et al. 2012) and PhyML version 3.0 (Guindon and Gascuel 2003), respectively. The 13 PCGs from the mitochondrial genomes of 71 species, which included 20 Ephemeroptera species, 27 Odonata species, 16 Neoptera species, 5 Archaeognatha species, and 3 Zygentoma species, were used to investigate the phylogenetic relationships. Among them, Archaeognatha and Zygentoma were used as outgroups. To select conserved regions of the nucleotide, each alignment was performed by Gblock 0.91b (Castresana 2000) using default settings.

The phylogenetic relationship based on BI and PhyML analyses (Figure 1) showed that Ephemeroptera was the basal group of Pterygota and that Odonata was a sister clade to Neoptera, as also shown in Zhang et al. (2008). Among Ephemeroptera, *Siphluriscus chinensis* was the most primitive clade as well as the results of Ogden et al. (2009), Li et al. (2014), and Zhou and Peters (2003).

**Figure 1.**
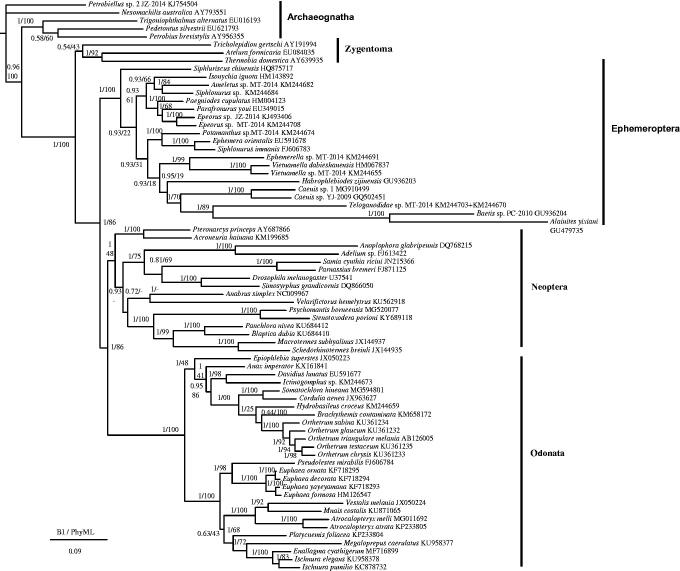
Phylogenetic tree of the relationships among 71 species of Pterygota based on the nucleotide dataset of 13 mitochondrial protein coding genes. The tree includes 20 Ephemeroptera species, 27 Odonata species, and 16 Neoptera species, as well as five Archaeognatha and three Zygentoma species as the outgroups (Clary et al. 1982; Nardi et al. 2003; Cameron et al. 2004; Yamauchi et al. 2004; Cook et al. 2005; Cameron et al. 2006; Podsiadlowski 2006; Stewart and Beckenbach 2006; Cameron and Whiting 2007; Carapelli et al. 2007; Zhang et al. 2008; Zhang et al. 2008; Comandi et al. 2009; Lee et al. 2009; Sheffield et al. 2009; Lin et al. 2010; Cameron et al. 2012; Jong et al. 2012; Li et al. 2014; Lorenzo-Carballa et al. 2014; Tang et al. 2014; Ma et al. 2015; Huang et al. 2015; Cheng et al. 2016; Feindt et al. 2016; Yang et al. 2016; Yong et al. 2016; Yu et al. 2016; Zhang et al. 2017; Zhang et al. 2017; Zhang et al. 2018). Numbers above the nodes indicate the posterior probabilities of BI and the bootstrap values of PhyML. The GenBank numbers of all species are shown in the figure.
